# Towards theoretically understanding how long-term memory semantics can support working memory performance

**DOI:** 10.1177/17470218241284414

**Published:** 2024-11-09

**Authors:** Rebecca Hart, Robert H. Logie, Louise A. Brown Nicholls

**Affiliations:** 1 Department of Psychological Sciences & Health, University of Strathclyde, Glasgow, UK; 2 Department of Psychology, University of Edinburgh, Edinburgh, UK

**Keywords:** Working memory, verbal memory, visuospatial memory, visual-spatial, semantic long-term memory

## Abstract

Working memory is the system that supports the temporary storage and processing of information. It is generally agreed that working memory is a mental workspace, with a combination of resources operating together to maintain information in mind for potential use in thought and action. Theories typically acknowledge the contributions of long-term memory to this system. One particular aspect of long-term memory, namely semantic long-term memory, can effectively supplement or “boost” working memory performance. This may be a relatively automatic process via the semantic properties of the stimuli or more active via strategy development and implementation. However, the precise mechanisms require greater theoretical understanding. In this review of the literature, we critically discuss theoretical models of working memory and their proposed links with long-term memory. We also explore empirical research that contributes to our understanding of the ways in which semantics can support performance of both verbal and visuospatial working memory tasks, with a view to potential intervention development. This includes the possibility of training people with lower performance (e.g., older adults) to use semantics during working memory tasks. We conclude that semantics may offer an opportunity to maximise working memory performance. However, to realise this potential, more research is needed, particularly in the visuospatial domain.

Working memory is the limited capacity cognitive system that simultaneously processes and maintains information over periods of seconds (e.g., Baddeley, 2012; Cowan, 2017; Logie, Camos et al., 2021). This ability is crucial for moment-to-moment functioning in everyday life, including processing novel information. Working memory theories propose links with long-term memory (e.g., Baddeley, 2012; Cowan, 1999; Ericsson & Kintsch, 1995; Logie, 1995, 2011). Indeed, one of the most significant developments in the Baddeley and Hitch (1974) framework of working memory was the modelling of “crystallised” resources, specifically, visual semantics, language, and episodic long-term memory, as interactive support for “fluid” working memory (Baddeley, 2000). Meanwhile, research considering the role of strategies during working memory task performance has been rapidly increasing (e.g., Gonthier, 2021; Logie, 2018a, 2023b; Morrison et al., 2016). This has highlighted that the way in which participants perform a task, including using semantics, can maximise their capacity. For example, during a working memory task, participants might construct a meaningful story with the memoranda, memorise abstract visual configurations based on meaningful prior knowledge, or assign meaningful labels to abstract stimuli (e.g., Forsberg, Johnson et al., 2020; Gonthier, 2021; Hardman et al., 2017; Souza & Skóra, 2017). Importantly, this is dissociable from the use of episodic long-term memory. To illustrate, semantic strategies involve the use of stored meaningful knowledge about the world, whereas using episodic long-term memory would involve activating memory traces from previous events, such as preceding trials of the same task (e.g., remembering the location of a target or a sequence of targets) to support recall for the current trial (Kemps, 2001; see Gonthier, 2021). Indeed, distinctions between episodic and semantic long-term memory are made in the theoretical literature. Tulving (1972) assumed that the experiences captured by episodic long-term memory are distinct from the general knowledge in semantic long-term memory. However, other theorists do not agree with this dichotomy. One perspective suggests that episodic and semantic long-term memory are end-points on a continuum of representations, running from contextually dependent representations of previously experienced events to the context-free representations of factual knowledge (Craik, 2020). In the present review, we focus on how specifically semantic information (i.e., general knowledge not tied to a particular event or context) is associated with working memory performance. Indeed, there is a large body of evidence showing superior performance on working memory tasks with meaningful versus abstract stimuli. This has been argued not to be due to accessing episodic information (Brady et al., 2016).

Over the years, working memory research has been focussed largely on the verbal domain. However, in the last three decades in particular, there has been a substantial increase in the volume of research in the visuospatial domain (for reviews, see Hakim et al., 2021; Liesefeld & Müller, 2019; Logie, 1995, 2011; Logie, Belletier et al., 2021; Luck & Vogel, 2013; Postle, 2021). Furthermore, one important aspect of the Baddeley and Hitch (1974) framework of working memory is the domain-specificity of verbal and visuospatial storage systems. Despite semantic long-term memory appearing to be a promising resource for supporting working memory across modalities, there remains a need for more substantial work that explores semantic effects in both verbal and visuospatial working memory. Therefore, this review is intended to make an important contribution to the literature by providing an integrated exploration of semantic effects in both working memory domains. Furthermore, this review will feature direct comparisons of the contributions of semantic long-term memory to verbal and visuospatial working memory tasks. This could provide important insights into how semantic long-term memory might interact with and benefit working memory.

We begin this review by highlighting how the Baddeley and Hitch (1974) framework of working memory proposed links with long-term memory while considering adaptations to this framework over time. We also consider alternative models of working memory, including perspectives associated with recent advancements in computational modelling. Then, empirical evidence from both classic and more contemporary studies that have explored the effects of semantic long-term memory on working memory performance will be reviewed across the verbal and visuospatial domains, in turn. Overall, this review uniquely compares and contrasts empirical evidence of semantic effects on both working memory modalities. Furthermore, we highlight outstanding issues and directions for future research on the topic.

## Theoretical models of working memory

Working memory can process and retain information for several seconds after it is no longer accessible via the environment, and capacity is limited by the amount and precision of what is to be remembered (e.g., Brown et al., 2006; Cowan, 2010; Logie, 2011; Luck & Vogel, 1997; Ma et al., 2014). Memory traces will also be lost rapidly due to decay (e.g., Barrouillet & Camos, 2021), interference (e.g., Lin & Oberauer, 2022), or both decay and interference (e.g., Cowan et al., 2021). Furthermore, with information encoded over time, the most recently presented stimulus will have priority within the focus of attention (Baddeley et al., 2021; Cowan et al., 2021).

Undoubtedly, the “multiple component” perspective, which has been built upon the original Baddeley and Hitch (1974) model, is one of the most influential theoretical frameworks of working memory. Multiple component models assume that working memory comprises several domain-specific systems that interact to support ongoing task performance. Baddeley and colleagues’ framework (e.g., Baddeley, 1986, 1996, 2000, 2007, 2012; Baddeley et al., 2021) consists of a phonological loop, visuospatial sketchpad, episodic buffer, and central executive. The phonological loop processes and temporarily retains a small amount of phonologically coded verbal material, whereas the visuospatial sketchpad supports the processing and temporary retention of visual and spatial properties of stimuli (e.g., colour, shape, orientation, location, movements; see also Logie, 1995, 2003, 2011). The system is controlled by central executive resources that coordinate the activities of the phonological loop and visuospatial sketchpad, although Logie (2016, 2023b; Logie, Belletier et al., 2021) and others (e.g., Hazy et al. 2006, 2007; Vandierendonck, 2016, 2021; Willshaw, 2006) have argued that executive resources might be implemented by distributed control rather than a single, central mechanism. The concept of an episodic buffer acts as an interface between long-term memory and the domain-specific components of working memory to retain a temporary, cohesive representation of combinations of verbal and visuospatial information, along with information derived from long-term memory, including semantics.

While we currently focus on the multiple component approach to working memory, particularly given the topic of this Special Issue, it is also important to acknowledge that there are other influential models. For example, one alternative theoretical framework, embedded processes, proposed by Cowan (e.g., 1999, 2016; Cowan et al., 2021, 2024), takes a more “top-down” approach, focussing on overall capacity of working memory and the control of limited capacity attention. Within this framework, working memory comprises incoming sensory information along with activated long-term memory, including the semantic properties of stimuli. A key component of this model is the limited capacity focus of attention, which highlights the mental representation of certain stimuli. Executive control can also actively refresh working memory contents and selectively retrieve task-relevant information from long-term memory. Importantly, there are no distinguishable stores or boundaries between stimulus modalities.

Within the time-based resource-sharing model (TBRS; e.g., Barrouillet et al., 2004; Camos & Barrouillet, 2018), a key feature is a central executive loop, which interacts with more peripheral systems, including episodic and declarative (semantic) long-term memory and sensory systems. Stimuli are vulnerable to temporal decay and representation-based interference, but degradation can be prevented through the focus of attention, which may process or refresh only one item at a time (see also Oberauer, 2002). Therefore, working memory must rapidly switch between processing and refreshing to complete tasks and avoid information loss. Similar to Baddeley and colleagues’ (e.g., 2021) multiple component approach, multimodal information including, for example, verbal, visual, and semantic content, may be stored in an episodic buffer.

Recently, attention has been given to the wide range of different theoretical conceptions of working memory that have arisen from a plethora of apparently contradictory empirical evidence since the seminal Baddeley and Hitch (1974) paper (for reviews, see Cowan, 2017; Logie, 2023a; Logie, Camos, et al., 2021). Recent theoretical advances, as described by Logie (2023a, 2023b; see also Cowan et al., 2020), have involved adversarial collaboration from working memory theorists, with the aim of resolving theoretical debates that have been ongoing for two or more decades without any clear resolution. The result has been that theoretical assumptions have been modified to become more similar. For example, Cowan (Cowan et al., 2014, 2021) has now acknowledged that there may be limited storage of domain-specific information separate from the focus of attention in peripheral storage components of working memory. Also, the TBRS model now includes multiple components in its framework, including a phonological, visuospatial, and motor buffer, as well as motor programmes for articulation (Barrouillet & Camos, 2021). Logie (2023a) suggests that rather than being mutually incompatible, these views of working memory may, in fact, be more similar than they seem while reflecting different levels of explanation or emphasis. In other words, the embedded processes model focuses on the overall capacity of working memory, while multiple component models focus on how that capacity is achieved. TBRS also originally focussed on overall capacity but more recently has shifted to consider different contributions to that capacity (Barrouillet & Camos, 2021).

Notably, computational modelling of working memory goes beyond verbal theories. It can predict, simulate, and explain previous findings from human data (Logie, 2018b). To date, there have been many computational investigations of working memory (e.g., Burgess & Hitch, 1999; Oberauer & Lewandowsky, 2011; Page & Norris, 1998), but most focus on a particular aspect of working memory functioning. For example, an area that has received considerable attention is serial recall. One view, named the primacy model (Page & Norris, 1998), argues that items are retained in memory with various levels of activation. The first item in a list is the most highly activated and recalled first, the second item is the next most highly activated and recalled second, and so on. Forgotten items do not have an effect on recall of later items, and transposition errors occur due to similar levels of activation for items in the middle of a list. Alternatively, there may be a separate learned representation of serial order where an item is associated with an external representation of position. Therefore, the first item would be associated with the external representation of the first list position, the second item with the second list position, and so on (e.g., Burgess & Hitch, 1999). Similarly, a computational implementation of the TBRS model was developed by Oberauer and Lewandowsky (2011), called TBRS*, which implements the verbal theory (Barrouillet et al., 2004). Associations between item and position are stored in a two-layer connectionist network. For each item, there is a node in the item layer that is connected with a set of markers in the position layer. These associations decay with time, which can be prevented through refreshing, via the focus of attention. Adjacent positions share a proportion of markers, simulating the tendency for people to make errors between adjacent positions, compared to other positions in the list. This has extended into further adapted models, which, for example, take into account both interference- and decay-based mechanisms of forgetting (Lemaire & Portrat, 2018). These are just some examples of the role and influence of computational models, which allow testing specific predictions and making theoretical developments in working memory (for reviews, see Logie, 2018, 2023b; Hitch, 2023).

## Theoretically linking working memory and long-term memory

Building on the original multiple component working memory model by Baddeley and Hitch (1974), Baddeley (2000) proposed the episodic buffer. This supports a temporary, multimodal representation for the current task and allows conscious access to the contents of working memory. Importantly, this multimodal storage system integrates information from different sources, including from long-term memory. Similarly, in the TBRS model (Barrouillet & Camos, 2014), working memory is distinct from long-term memory. Yet, the process of refreshing memoranda using attentional resources can draw upon semantic knowledge from long-term memory to reconstruct decaying memory traces in the episodic buffer.

Logie (2011, 2016; Logie, Belletier et al., 2021) argued that the concept of the episodic buffer may be viewed as arising from interactions between domain-specific components and long-term memory rather than from a separate component. Specifically, Logie (e.g., 1995, 2003, 2011; Logie, Belletier et al., 2021) proposed that perceived information activates stored knowledge about the stimuli, including semantic information, and that activated information is then processed and maintained by interacting working memory components. Logie (2011, 2016, 2023b) views the episodic buffer as a convenient label for the dynamic interaction between components of cognition, including temporary storage in the phonological loop and visual cache, and currently activated semantic and episodic information in long-term memory. Thus, these interconnections allow for aurally presented stimuli (verbal or nonverbal sounds) to be encoded visually, and for visually presented stimuli (shapes and objects, or words and letters) to be encoded verbally, in each case along with their semantic associates. This is consistent with Paivio’s (1971, 1991) dual-coding theory in which both imagery and verbal codes may represent information. From that perspective, two types of code are processed by two distinct systems, creating separable but linked representations. Therefore, both imagery and verbal labels can be accessed from stored knowledge in long-term memory to aid temporary storage and recall of information. Importantly, the ability to code a stimulus across multiple resources (i.e., attaching both imagery and verbal codes) will vary by stimulus and, where possible, elaborates meaning and can enhance recall.

In the embedded processes model, working memory is viewed as a subset of activated long-term memory (Cowan, 1999, 2016). If the sensory features of a perceived item have been encountered before, then portions of long-term memory are activated. Several items may be further analysed through the focus of attention, which interacts with long-term memory to extract associated meaning. Therefore, rather than assuming that working memory and long-term memory are two separate systems that can interact, embedded processes views a key component of working memory as temporarily activated information in long-term memory that has been made accessible through the focus of attention. In other words, working memory and long-term memory are different modes of operation for the same system: what is active and in the focus of attention for the current task, and what is not active or required for the current task.

There have been several advancements in computational modelling to specifically consider semantic memory in working memory functioning. Some models assume that the meaning of a word is derived from averaging its use across contexts (e.g., Jones & Mewhort, 2007). However, other models argue that memory is a single system that records specific instances and general knowledge about word meaning emerges spontaneously from episodic memory during retrieval (e.g., Jamieson et al., 2018, 2022). Therefore, these instance-based models can account for the subordinate meanings of words in appropriate contexts (Jamieson et al., 2018). For example, being able to construct the meaning for the word “bank” (which can have several meanings depending on the context) from the activation of exemplars that match the way in which the word is being used (Jamieson et al., 2022). Furthermore, the computational TBRS* model (Oberauer & Lewandowsky, 2011) originally did not consider semantic effects in working memory. However, as this model has been shown to successfully reproduce other well-established working memory effects, it may be a useful architecture to model semantic effects (Kowialiewski et al., 2021). Indeed, an adaptation of this architecture, called TBRS*-S (semantic), accounts for semantic similarity effects in working memory (Kowialiewski et al., 2021). This model includes a separate long-term memory layer in which items can be directly activated. Semantically related items can reactivate each other in long-term memory, and attentional resources can then be reallocated for maintenance purposes.

Importantly, working memory capacity is assumed to be limited to around 3–5 chunks of information (Cowan, 2010), but this can be boosted using different strategies (Logie, 2018a, 2023b), such as drawing upon long-term memory resources (Ericsson & Kintsch, 1995; Hambrick et al., 2021). Specifically, semantics (i.e., meaningful conceptual and factual world knowledge stored in long-term memory) can free up (Kowialiewski & Majerus, 2020; Kowialiewski et al., 2021) or supplement working memory resources. Using semantic resources to support working memory allows individuals to develop exceptional abilities such as simultaneously playing several games of chess or memorising thousands of digits of pi (Ericsson & Moxley, 2014; Ericsson et al., 2018). To illustrate, performance on immediate verbal recall tasks is substantially larger for words presented within a meaningful sentence, compared with random word lists (e.g., Allen et al., 2018; Baddeley et al., 2009; Brener, 1940). This impact of stored knowledge can be explained via a process of redintegration (Hulme et al., 1991; Walker & Hulme, 1999). Degraded phonological forms of verbal material from working memory can be reconstructed (i.e., redintegrated) based on semantic cues that prompt retrieval of information from long-term memory (Poirier & Saint-Aubin, 1995; Saint-Aubin & Poirier, 1999). Schweickert’s (1993) multinomial processing tree model of redintegration has been used to explain the effect of stored knowledge in long-term memory on working memory recall (e.g., Gathercole et al., 1999; Saint-Aubin & Poirier, 1999). This model argues that there are two possible routes for successful recall of an item in memory: directly accessing an intact memory trace or reconstructing a degraded memory trace. Greater availability of semantics within memoranda can aid reconstruction. In other words, if the degraded trace is easier to reconstruct through higher-level processes (e.g., using semantics), then the probability of successful recall is greater.

Dominant working memory theories, therefore, generally agree that working memory draws upon, or even depends, to some extent, on activated long-term memory. However, the precise mechanisms by which working memory and long-term memory interact remain to be fully understood. In the following sections, empirical evidence regarding the impact of semantics on working memory performance will be reviewed, considering the verbal and visuospatial domains in turn.

## Semantics to support verbal working memory

Theoretically, semantics may be automatically activated at perception (Cowan, 1999, 2016; Logie, 1995, 2011; see also Campoy et al., 2015), particularly in a working memory task with meaningful stimuli or, in other words, featuring high semantic availability. Research has investigated the effects of the semantic properties of verbal stimuli, including lexicality, meaningfulness or concreteness of words, and relatedness across items. Greater availability of semantics increases the number of items that can be recalled (e.g., Campoy et al., 2015; Kowialiewski & Majerus, 2018, 2020; Loaiza & Camos, 2018).

In line with multiple component models, phonological coding clearly plays a fundamental role in verbal working memory performance (Conrad, 1964; Conrad & Hull, 1964). For example, lists of short words are better recalled than lists of long words (e.g., Baddeley et al., 1975). This has been most widely interpreted as arising because long words take longer to rehearse and keep active in working memory, and the representations decay faster. The maintenance of verbal material in working memory is also thought to depend on interactions with stored linguistic knowledge in long-term memory (Majerus, 2019). Indeed, items stored in working memory can be represented at different levels, such as phonological versus semantic (Cowan et al., 2022; Shivde & Anderson, 2011). Evidence for separable buffers for phonological and semantic information also comes from patient studies. For example, individuals with semantic working memory deficits show typical phonological effects on capacity, whereas those with phonological deficits do not. Similarly, those with phonological deficits show typical semantic effects (e.g., lexicality effect), whereas those with semantic deficits do not benefit from the semantic properties of words (Martin et al., 2021).

### Words versus non-words

Perhaps the most commonly observed semantic effect in verbal working memory is superior recall for words versus non-words. For example, Hulme et al. (1995) presented lists of words and non-words to manipulate the representations of items in long-term memory and found that memory span for immediate recall tasks was significantly higher for words than for non-words (see also Camos et al., 2019). It was argued that this benefit reflects contributions of semantic long-term memory to task performance (see also Hulme et al., 1991; Loaiza, Duperreault, et al., 2015). Similarly, Loaiza, Rhodes, et al. (2015) manipulated the meaningfulness of to-be-remembered words, including words that young adults would or would not be likely to know (e.g., current vs outdated). Participants were better at recalling the known versus unknown words. This suggests that participants were able to encode and use information from long-term memory to improve recall for the known words. However, the lexicality effect may not be completely attributable to semantic long-term memory. Indeed, memory for words is also influenced by phonotactic knowledge, that is, phonological knowledge about phoneme/syllable transition probabilities (e.g., Gathercole et al., 1999; Majerus et al., 2004). Importantly, dissociations have been reported between phonological and semantic effects during verbal working memory tasks (e.g., Nishiyama, 2013, 2018). For instance, articulatory suppression does not remove the benefit of higher semantic availability. This suggests that semantic representations can operate separately, without relying on phonological representations or articulatory rehearsal (e.g., Poirier & Saint-Aubin, 1995; Romani et al., 2008).

### Semantically related versus unrelated words

Semantic effects have also been observed for recalling lists of related versus unrelated words. Baddeley (1966a) investigated immediate serial recall of lists of five words, which were repeatedly drawn from either a set of eight related or unrelated words. Participants listened to the word lists and, immediately after, were asked to recall the words in the order in which they were presented. Importantly, the words were written on cards and visible to participants throughout the session, so the focus was on order of recall. Results showed a clear effect of phonological similarity between words for recall, with phonologically similar words recalled less accurately than phonologically different words. However, a small and inconsistent effect of semantic similarity existed, in which related words were recalled less accurately than unrelated words. In this case, semantically similar words were believed to have less distinctive traces in working memory, impairing the use of semantic information to support recall. It has since been highlighted that the study paradigm encouraged phonological retention of the words, as the sequences were repeatedly drawn from the same pool of words and participants were able to view these continuously. Therefore, the focus on the retention of serial order of presentation most likely encouraged participants to code the words phonologically and to ignore the semantic information, which was less useful in this context (e.g., Ishiguro & Saito, 2021; Romani et al., 2008). Furthermore, Baddeley (1966b) found a negative effect of semantic similarity on recall when memory was tested following a 20-minute delay period, emphasising the contribution of semantics to long-term recall.

Conversely, when words are drawn from an open set (i.e., choices are not limited to a small set of words), semantically related word lists are more accurately recalled than unrelated words. Tse (2009) administered to-be-remembered word lists, which were either from the same or different semantic categories or associations. Lists from the same semantic category (e.g., fruit; apple, banana) or association (e.g., sweet, sour) were better recalled than unrelated word lists (see also Aka et al., 2021; Channon & Daum, 2000; Poirier & Saint-Aubin, 1995; Saint-Aubin et al., 2005; Saint-Aubin & Poirier, 1999). To expand, immediate recall for word lists is highly cue-dependent (Kahana, 1996). Word recall may, therefore, automatically activate semantic cues in long-term memory, prompting retrieval of related words (Howard & Kahana, 2002). This may also be explained by the redintegration perspective (Poirier & Saint-Aubin, 1995; Saint-Aubin & Poirier, 1999).

### Concreteness

A significant body of research has found a concreteness effect, whereby words that are more concrete or imageable (e.g., jacket, pencil) are better recalled than words that are more abstract or non-imageable (e.g., jealous, peace; Paivio & Csapo, 1969; Postman & Burns, 1974; Taylor et al., 2019). In verbal working memory paradigms, the concreteness effect has been observed using a variety of tasks including serial and cued recall (e.g., Acheson et al., 2010; Bourassa & Besner, 1994; Richardson, 2003; Romani et al., 2008; Walker & Hulme, 1999). Clinical studies have also found that, when people who struggle with articulatory rehearsal are asked to recall words, they still benefit from high imageability (Howards & Nickels, 2005). Furthermore, the neural representation of concrete nouns has been identified from functional magnetic resonance imaging (fMRI) data. For example, using this method, Just et al. (2010) revealed three semantic factors that underpinned the neural representation of concrete nouns, namely manipulation (can it be held and manipulated?); eating (is it food-related?); and shelter (can it be used for shelter?). Each factor was represented across three to four locations in the brain that correspond with areas activated during non-linguistic tasks. These investigations extend understanding of the representation of concepts in the brain, as well as allow decoding of information from these activation patterns.

One explanation for the concreteness effect is that concrete words readily have both visual and verbal representations, while abstract words typically have only verbal representations (i.e., dual-coding theory; Paivio, 1991). As a result of being processed in both verbal and visuospatial systems, and therefore having two possible representations for retrieval, concrete words are retrieved more easily. Another explanation is the context availability hypothesis, where retrieval of concrete words is supported by access to contextual information from prior exposure, including semantic information about the meaning of the word and situations where it may appear (Schwanenflugel & Shoben, 1983). Indeed, the concreteness effect has been found to disappear when contexts are provided for both concrete and abstract words (Schwanenflugel & Shoben, 1983).

Similarly, immediate serial recall of digits is improved when the digits are presented via a meaningful spatial keypad configuration versus serially in a single location, a phenomenon termed visuospatial bootstrapping (e.g., Calia et al., 2019; Darling et al., 2017). This further indicates a role for multimodal representations in working memory, including a role of familiarity specifically at encoding (Allen et al., 2015, 2023; Darling et al., 2012, 2014, 2017), which may be relatively automatic (Calia et al., 2019). Interestingly, Allen et al. (2023) recently found that articulatory suppression during the task maintenance period increased the size of the visuospatial bootstrapping effect (see also Romani et al., 2008, for a similar effect with concrete word recall). The effect was reduced or abolished with administration of attention-demanding or spatially oriented tapping tasks during maintenance. This suggests interacting roles for spatial and, to some extent, attentional processing resources for the semantic effect during verbal working memory, at least during maintenance.

### The strategic use of semantic long-term memory in verbal working memory

A strategy is a procedure, or set of procedures, that an individual can use when performing cognitive tasks (Lemaire, 2016; Logie, 2018a, 2023b; Logie et al., 1996) and that can impact working memory performance (e.g., Belletier et al., 2023; Bengson & Luck, 2016; Brown & Wesley, 2013; Dunlosky & Kane, 2007; Gonthier & Thomassin, 2015; Morrison et al., 2016). In fact, despite the known capacity limits of working memory, tasks that are designed to measure working memory span may instead reflect variations in strategy use, strategy efficiency, and strategic adaptation during task performance (Logie, 2023b). Theoretically, Logie’s (1995, 2011) model incorporates activation of relevant semantic knowledge from long-term memory that contributes to working memory. This may help to account for different interpretations of earlier findings. For example, in some conditions, such as recalling short sequences of unrelated verbal material (e.g., Baddeley, 1966a), a phonological strategy may predominate. However, an individual may switch to a semantic strategy, depending on task properties and priorities (Richter et al., 2015) and their own strategy “repertoire” (Lemaire, 2016).

Self-reporting strategy use has been a useful way to show the extent to which participants use different strategies during verbal working memory tasks. The importance of strategy in working memory was highlighted by Logie et al. (1996) where, upon completion of verbal working memory tasks, participants’ strategic approach was queried. Participants who reported employing a semantic strategy showed weaker effects of word length and phonological similarity of words than those who reported using a phonological strategy (see also Hanley & Bakopoulou, 2003). Studies have since investigated retaining episodic events in long-term memory and have found that strategically using pre-existing semantic knowledge to enrich new information (i.e., elaboration) is a beneficial approach (e.g., Bartsch et al., 2018; Bartsch & Oberauer, 2021). Elaboration is the process of enriching memory representations by activating meaning and linking it to stored semantic associations (Craik & Tulving, 1975). There is less evidence regarding the use of an elaboration strategy to support working memory. Although, self-reported elaboration strategies have been linked to better recall. Dunlosky and Kane (2007) asked participants to provide set-by-set reports of strategy use during an operation span task involving a series of calculations, each followed by a word for later recall. Following attempted recall of the to-be-remembered words of a given set, participants reported the strategy, if any, that they used to complete the task. Participants were asked whether they: read each word as it appeared; repeated the words; generated a sentence to link words; developed mental images of the words; meaningfully grouped words; or did something else. The researchers then compared a composite score derived from the normatively effective strategies (imagery, sentence generation, and grouping) with that from the two or more passive strategies. Span was significantly greater for normatively effective strategies. More recently, Belletier et al. (2023) observed that certain reported strategies (e.g., forming links between the memoranda using prior knowledge) were positively associated with verbal working memory performance. Clearly, in this case, there is a range of potential working memory strategies that draw upon a variety of cognitive resources, and using semantic long-term memory is one of these.

## Semantics to support visuospatial working memory

Although there is a large literature on the use of semantic knowledge in visual imagery (e.g., Kosslyn, 1994; Paivio, 1971; Pearson & Kosslyn, 2015), considerably less research has investigated the effects of semantic knowledge specifically on visuospatial working memory. However, the volume of research in this domain has been increasing. It is now generally accepted that pre-existing semantic representations can also support visuospatial working memory performance (see Chung et al., 2023a, for a review).

### Concreteness

Similar to research within the verbal domain, a concreteness effect has also been observed in visuospatial working memory performance. Enhancements have been found for recall when meaningful verbal labels are provided alongside a visual stimulus. For example, Verhaeghen et al. (2006) asked participants, who were non-readers of the Chinese language, to remember Chinese characters. They provided verbal labels (concrete nouns) alongside the characters, which improved character recognition. It was argued that the meaningful labels increased the imageability/concreteness of the stimuli, recruiting supportive semantic representations in long-term memory that were assimilated to the characters. Specifically, Chinese characters that were presented alongside highly imageable words were better remembered than characters that were presented with less imageable words. Furthermore, for the Chinese characters paired with imageable words, there was an additional benefit when the words were familiar. Overall, the associations made between the Chinese characters and the verbal labels appear to be mostly visual in nature and benefit further from familiarity.

Familiarity also boosts memory specifically for faces (Jackson & Raymond, 2008; Young & Burton, 2017). One explanation is that it is the semantic knowledge associated with familiar faces that facilitates memory. However, there are alternative accounts for this effect. For instance, it has been proposed that it is easier to generate a verbal label for a familiar versus an unknown face, which will strengthen the representation in memory (i.e., dual-coding theory; Paivio, 1991). Yet, studies have found that verbal rehearsal is not required for this benefit to be observed. For example, using a change detection task, Jackson and Raymond found that participants demonstrated significantly better performance for famous versus unfamiliar faces. This advantage for famous faces was not affected by a verbal suppression task, suggesting that it was not due to strategic incorporations of verbal working memory/articulation. However, face inversion abolished the famous face advantage, suggesting that performance was enhanced by the existing representations of famous faces in long-term memory. These findings may be limited in their generalisability. However, the results extend to situations that compare familiar and unfamiliar objects from various categories. For example, Starr et al. (2020) found that performance of an immediate test of memory was significantly better for familiar, real-word objects than for unfamiliar, obscure objects (see also Brady et al., 2016; Brady & Störmer, 2022; Torres et al., 2023). This benefit was still observed when participants performed a concurrent verbal task to inhibit phonological coding of items. Indeed, it has been demonstrated that recognising the stimulus as meaningful is the source of the benefit for these stimuli and not physical differences between stimuli (Asp et al., 2021). Furthermore, Loaiza et al. (2023) suggested that the observed concreteness effect in visual working memory operates independently of attention (see also Logie, 1995, 2003, 2011).

Prior knowledge about object-colour associations also improves visual working memory for colours. Sobrinho and Souza (2023) investigated whether presenting a colour with a congruent object (e.g., yellow, banana) at encoding would boost participants’ visual working memory performance. The congruency between the colours and objects was thought to allow participants to use long-term memory for objects to facilitate the storage of the relevant feature (i.e., colour) in visual working memory. However, colour-item congruency and, thus, prior knowledge only benefitted performance when it was relevant to the task (i.e., when both object and colour were test-relevant). Other studies have found that working memory performance for low-level features (i.e., colour) is increased if they are encoded in a meaningful way. Specifically, colours are better remembered when they are presented as part of meaningful objects, which allow high-level features to serve as a scaffold for associated lower-level features (Chung et al., 2023c). Importantly, colours were randomly assigned to colour-neutral objects to control for the involvement of episodic long-term memory for specific, coloured objects.

Considering concreteness in terms of spatial tasks, expert chess players have been found to be able to store a higher number of meaningful chess patterns than novice chess players but only for legitimate configurations. This clearly implicates stored knowledge in long-term memory (Gobet & Simon, 1996). Similarly, using a visual *n*-back task, in which participants are asked to match a currently presented item with an item presented *n* trials previously, Rudner et al. (2016) found that sign language users performed more accurately with their own sign language compared to another sign language. In both examples, individuals were able to draw upon pre-existing semantic representations to enhance visuospatial working memory performance.

### The use of semantic long-term memory in visuospatial working memory

**Figure 1.**
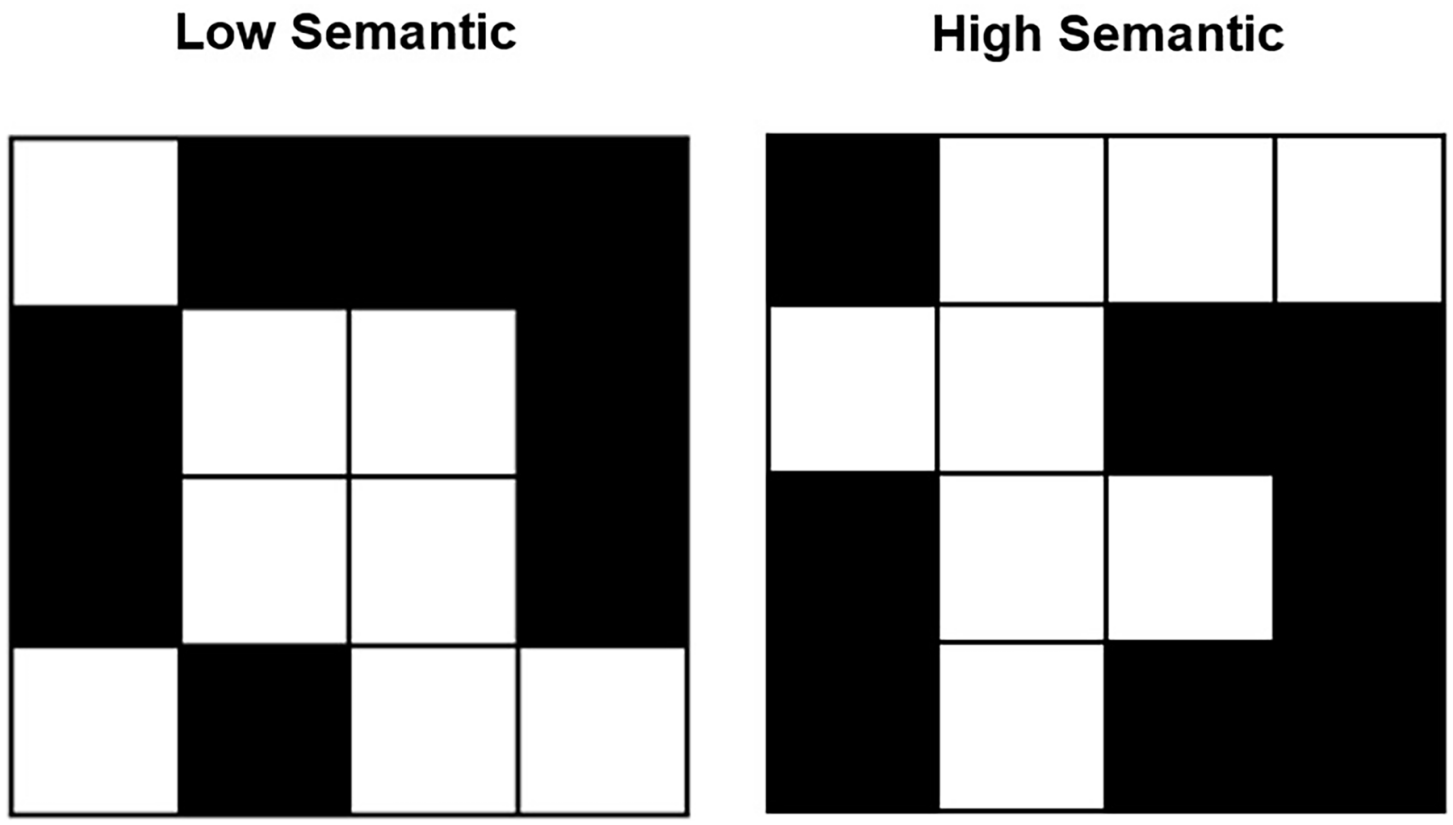
Example low and high semantic stimuli from the modified Visual Patterns Test (Brown et al., 2006). Participants identified more meaningful shapes within the black or white cells in the high semantic stimuli. In this example, an “i,” “back-to-front c,” or an “F” are some of the meaningful patterns that participants could identify. These stimuli are taken from task level of complexity 8 (8 black cells for recall).

The impact of semantics on visuospatial working memory performance has been investigated using a variety of tasks that can be more demanding on visual or spatial resources (Logie, 2011). However, one popular method for investigating the role of semantics in visuospatial working memory is to use visual matrix tasks (e.g., Beigneux et al., 2007; Johnson et al., 2010; Logie & Pearson, 1997; Orme, 2009; Phillips & Baddeley, 1971; Phillips & Christie, 1977; Riby & Orme, 2013; Williamson et al., 2011). During the Visual Patterns Test (VPT; Della Sala et al., 1999, 1997; Wilson et al., 1987), participants view increasingly complex black-and-white chequered patterns and are asked to recall the pattern after a short delay. Based on participants’ ability to label configurations within the patterns in the original task, Brown et al. (2006) created “low semantic” and “high semantic” task versions. The most abstract (i.e., low semantic) and the more meaningful/verbalisable (i.e., high semantic) patterns available at each level of complexity were selected for each new task version (for examples, see Figure 1). This approach helps to control stimulus complexity while allowing investigation of the availability of semantics within the visual patterns.

Indeed, matrix patterns are typically better recalled when they are higher in semantic availability or, in other words, contain more potential meaning or familiarity (e.g., Brown et al., 2006; Hamilton et al., 2018; Riby & Orme, 2013). This is because, upon perception of a meaningful visual stimulus, semantics can be automatically activated (Logie, 1995, 2003, 2011; see also Orme et al., 2017; Plaska et al., 2021). However, importantly, semantics can also be strategically derived through actively searching the stimulus for meaning (Logie, 1995, 2011). Greater semantic availability in matrix patterns improves change detection accuracy and processing speed (Mammarella et al., 2014; Riby & Orme, 2013) and benefits overall recall, at least in young adults and typically developing children (Brown et al., 2006; Brown & Wesley, 2013; Hamilton et al., 2018; Nicholls & English, 2020). Orme and colleagues (Orme et al., 2017; Riby & Orme, 2013) also provided neuroimaging (event-related potential/ERP) evidence showing that high semantic stimuli are associated with less early-stage visual processing and lower memory encoding load, due to the activation and involvement of long-term knowledge. This was alongside the later stage, more active visual working memory processing, presumably to combine and maintain both the semantic and visuospatial content (see also Bor et al., 2003). Similarly, other experiments that have recorded neural activity using the electroencephalogram (EEG) have demonstrated that delay activity during the retention period is higher for meaningful versus abstract stimuli (Asp et al., 2021; Brady et al., 2016).

In line with this neuroimaging evidence, and in the context of the automatic activation of semantics, in a high semantic task, participants may require less processing-intensive resources to encode and retain the visuospatial details. However, processing-intensive resources are still likely involved in both tasks but in different ways. For example, Brown and Wesley (2013) found that the benefit of semantic availability in the modified VPT was removed with administration of an executive suppression task (random tapping; e.g., Darling et al., 2007; Vandierendonck et al., 1998). This suggests that there can be a cognitive cost to combining modalities, due to the requirement to associate and/or rehearse semantic representations in the context of the specific visual pattern (Brown & Wesley, 2013; Riby & Orme, 2013). Therefore, while semantic representations may be automatically activated upon perception of a meaningful stimulus, central executive resources appear to be required to be able to use and benefit from the semantics. Specifically, this seems to be the case when using multimodal coding (i.e., visual + verbal or semantics) regardless of the way in which semantics have been activated (i.e., automatic versus more actively/strategically derived). Use of a semantic strategy may be executively demanding, especially if active refreshing of representations is required over a period of time. However, this approach could help to reduce the memory load associated with more challenging, abstract configurations and reduce the resources required for both early-stage processing and retrieval.

A few studies to date have assessed self-reported strategies during visual matrix task performance, showing associations between strategy use and task capacity (e.g., Brown & Wesley, 2013; Hart & Nicholls, 2024; Nicholls & English, 2020). For example, Brown and Wesley found that young adults who reported combining visual and verbal strategies when completing both tasks from the modified VPT (Brown et al., 2006) achieved better memory performance (see also Souza & Skóra, 2017). Importantly, and counterintuitively, the “combiners” outperformed “non-combiners” on the low semantic but not the high semantic task version. However, this aligns with theory. When there was greater semantic availability, specifically the non-combiners’ performance was boosted relative to low semantic task performance. In contrast, the more efficient combiners were able to perform as well on the low semantic task as they did on the high semantic task, presumably due to strategically creating meaningful configurations in the more challenging, abstract patterns. Furthermore, Hart and Nicholls found that use of semantics during the modified VPT (Brown et al., 2006) was a frequently reported strategy. Reflecting the different ways in which semantics can be used, participants were queried on the extent to which they: 1) automatically noticed meaningful or familiar shapes (e.g., symbols, animals, etc.) within the patterns (i.e., without trying); 2) actively tried to find or look for meaningful or familiar shapes within the patterns; and 3) used meaningful or familiar information to remember the patterns, regardless of how that information was initially noticed. Participants reported using semantic strategies at least sometimes during the task and, importantly, some positive associations were observed with performance. Notably, this was subject to task instructions and individual differences. For example, in this study, older adults did not benefit from higher semantic availability in the patterns and also used a less efficient strategic approach. Promisingly, use of semantic strategies was shown to be positively associated with older adults’ performance, but specifically for more meaningful, high semantic patterns for which semantic codes are more readily available and more likely to be automatically activated (Forsberg et al., 2019; Nicholls & English, 2020). Similarly, Ozimič et al. (2023) interviewed participants regarding their strategy use during a change detection task. Many strategies were reported, one of which was pattern recognition. This was most often reported by participants when encoding positions and involves immediately becoming aware of a pattern within the stimuli. Qualitative analysis revealed that this helps to account for the target stimuli together, with one participant reporting that it was the perceived familiarity of the pattern that made it easy to remember. This demonstrates the involvement of semantic long-term memory.

Notably, verbalisation and semantic availability are clearly highly related (e.g., Lewis-Peacock et al., 2015). However, several studies have shown that articulatory suppression does not remove the benefit associated with high semantic availability in visual matrix patterns or in multimodal stimuli more generally (Brady et al., 2016; Brady & Störmer, 2022; Brown & Wesley, 2013; Chung et al., 2023b; Delogu et al., 2009; Orme, 2009; Plaska et al., 2021). Activated semantics is, therefore, the most likely source of the benefit. In other words, if a semantic concept is activated, verbal rehearsal is not needed for a benefit to be observed (Plaska et al., 2021). Long-term representations, therefore, likely augment the temporary visual representation and reduce visual noise (Forsberg, Johnson, et al., 2020; Hardman et al., 2017; Souza & Skóra, 2017). This is supported by neural evidence showing increased delay activity in parietal-occipital cortices for meaningful versus abstract stimuli, consistent with engagement of visual, and not language, areas of the brain (Asp et al., 2021; Brady et al., 2016).

## Semantic effects across verbal and visuospatial domains

This review has discussed well-established semantic effects in working memory across both the verbal and visuospatial domains. The majority of these investigations have been carried out in the verbal domain, revealing the influence of semantics in terms of lexical factors (e.g., words versus non-words, concreteness). These semantic manipulations have also been carried out in the visuospatial domain and, although this research is more limited, investigations have yielded similar findings. For example, visually presented, familiar, real-world objects are easier to remember than unfamiliar, obscure objects (Starr et al., 2020). Importantly, more research is needed to obtain a clearer pattern of the convergence between semantic effects in verbal and visuospatial working memory tasks and expand existing theoretical models. Currently, semantic effects are commonly discussed separately for verbal and visuospatial working memory in the literature. However, the effect of semantics across domains may be more coherent than has previously been assumed. Indeed, models have proposed the existence of a pan-modal integrative semantic region in the brain, which draws upon input from modality-specific knowledge brain regions to form deeper, transmodal representations (Hoffman et al., 2018). To simulate damage to this region, models have removed connections, showing impairments to activating associated information for concepts (Rogers et al., 2004). This is in line with multimodal deficits observed in patients with semantic dementia (Hoffman et al., 2018).

Importantly, in order for research to establish a clearer picture of the effect of semantics across verbal and visuospatial working memory tasks, greater clarification of what is precisely meant by “semantics” is required. Theoretically, it may be assumed that, like phonological similarity, semantic similarity is facilitative to item memory but detrimental to order memory. However, studies have found a facilitative effect of semantic similarity on serial recall performance (Kowialiewski & Majerus, 2020; Kowialiewski et al., 2021; Neath et al., 2022; Poirier & Saint-Aubin, 1995). As argued by Ishiguro and Saito (2021, 2024), these discrepancies in the similarity advantage are difficult to understand, because studies have manipulated semantics differently under the definition of “semantic similarity” (Tse, 2010; see also Neath et al., 2022). Most commonly, semantically similar word lists have been constructed based on category membership (e.g., Kowialiewski & Majerus, 2020; Poirier & Saint-Aubin, 1995; Saint-Aubin & Poirier, 1999; Saint-Aubin et al., 2005). In these studies, semantic effects have been attributed to using the shared category as a retrieval cue to aid memory for words in the list. Studies have also created semantically similar word lists based on word association (e.g., Kowialiewski & Majerus, 2020; Kowialiewski et al., 2021; Tse et al., 2011). In these studies, semantic effects may be explained by spreading activation to other associated words upon retrieval of a word. In a meta-analysis of previous studies investigating the semantic similarity effect in serial recall, Ishiguro and Saito (2021) argued that semantic similarity is detrimental to serial recall, whilst semantic association contributes to a facilitative effect. Overall, future studies should be clear about what facet of semantics is being manipulated so that we can correctly interpret the influence of semantic similarity effects (Ishiguro & Saito, 2024). Ultimately, this could help us to progress to a greater understanding of the mechanisms of using semantic memory in various tasks and help to inform the development of potential interventions.

## Implications and future directions

As outlined in this review, dominant theories of working memory propose important links with long-term memory. The working memory system can integrate information from different sources, including semantic knowledge stored in long-term memory. It is generally agreed that semantic knowledge can help to reconstruct decaying memory traces in the episodic buffer. However, important individual differences regarding the benefit of semantics appear to exist, and the precise mechanisms of using semantics during working memory tasks requires greater clarity.

### Individual differences in the strategic use of semantics

In young adults, certain autistic traits (e.g., attention to detail or focused visual processing) have been positively associated with visual working memory for novel objects (Richmond et al., 2013), including the low semantic version of the VPT (Nicholls & Stewart, 2023). This aligns with the assumption that this low semantic task relies more exclusively on visuospatial working memory resources (Brown et al., 2006). Furthermore, Mammarella et al. (2014) found a semantic benefit in the modified VPT for non-autistic children only. However, autistic children outperformed non-autistic children in the smaller-sized high semantic stimuli, likely because of their focussed visual processing style (see also Torenvliet et al., 2024).

Ageing is another factor that has received considerable attention in recent years (e.g., Brown et al., 2017; Johnson et al., 2010; Logie & Maylor, 2009; Swanson, 2017; see reviews in Logie & Morris, 2015). Despite reductions in working memory, the retrieval of meaningful information from long-term memory is relatively well preserved with healthy ageing (Naveh-Benjamin & Cowan, 2023). However, the evidence is mixed regarding the extent to which high semantic stimuli benefit older adults’ working memory performance. For example, higher semantic availability has been found to benefit older adults more than (Forsberg et al., 2019; Forsberg, Johnson et al., 2020), to the same extent as (Nicholls & English, 2020; Torenvliet et al., 2024), or even less than (Hamilton et al., 2018; Hart & Nicholls, 2024) younger adults. However, older adults may be able to use meaningful prior knowledge together with executive resources to actively encode stimuli in a meaningful way, to help boost memory (Naveh-Benjamin & Cowan, 2023; see also Reuter-Lorenz & Park, 2014). In verbal working memory, for example, young and older adults have been shown to use normatively effective strategies, including semantics, to the same extent (e.g., Bailey et al., 2009; Chevalère et al., 2020). In the visual domain, compared to younger adults, older adults report relying more exclusively on the more “obvious” strategy of visual rehearsal during visual matrix tasks (Nicholls & English, 2020). This suggests a need for greater understanding of the potential for semantic strategies to enhance working memory performance, particularly in certain groups.

### Semantic strategy training

Individuals, including groups who typically show a marked decline in some (but not all) aspects of working memory performance (e.g., older adults; Brown et al., 2017; Johnson et al., 2010; Hart & Nicholls, 2024; Logie & Maylor, 2009; Nicholls & English, 2020; Swanson, 2017) can be trained to use specific strategies. These individuals have, in some cases, demonstrated boosted performance following strategy training (e.g., Allen et al., 2021; Atkinson et al., 2018; Bailey et al., 2014; Forsberg, Fellman et al., 2020; Osaka et al., 2012; Rhodes et al., 2019). This shows promising initial evidence for the potential of strategy training to enhance working memory capacity. Yet, there is mixed evidence regarding training people to employ a semantic strategy. For example, training participants to organise random word lists into meaningful semantic categories has been shown to improve recall performance (Miotto et al., 2013, 2020). Campoy and Baddeley (2008) directly investigated the effects of strategies on verbal working memory performance via a strategy instruction procedure during an immediate recall task. Participants were instructed to memorise lists of phonologically similar versus dissimilar words, or short versus long words, using either a phonological or a semantic strategy. Participants instructed in a phonological strategy showed the typical phonological similarity effects, but these effects were not present in those instructed to use a semantic strategy. In addition, word length effects appeared in participants who were instructed to use a phonological strategy, whereas this effect was reversed for those instructed to use a semantic strategy. This reverse word length effect was argued to be because the longer words were more semantically related than the shorter words. Other studies have instructed elaborative strategies, such as mental imagery and sentence generation, but in some cases, this has not benefitted working memory performance (Bailey et al., 2009; Bartsch et al., 2019; Bartsch & Oberauer, 2021; Turley-Ames & Whitfield, 2003). It may be that elaboration (e.g., sentence generation) can involve processing additional irrelevant material, possibly affecting retrieval of relevant information (Bartsch & Oberauer, 2021). This could particularly be the case for those with lower pre-training performance (Turley-Ames & Whitfield, 2003).

Evidence is much more limited in the visuospatial domain. Hart and Nicholls (2024) administered semantic strategy instructions to try to boost young and older adults’ visual working memory performance. There was no effect of instruction on performance for either age group. However, regardless of age, those who received the semantic strategy instructions reported more active searching for familiar shapes and overall use of semantics compared to the control group. Promisingly, in instructed older adults and in the high semantic task only, use of semantic strategies was positively correlated with performance. This suggests that strategy instruction may encourage a more efficient strategic approach in older age, but this only benefits memory for more meaningful patterns, where semantic codes are more easily identified or created. Visuospatial working memory is integral to guiding our moment-to-moment functioning in everyday life. For example, behaviours such as navigation or driving a car are heavily reliant on visuospatial abilities (Guest et al., 2015; Underwood, 2007), including visuospatial working memory (Chaparro et al., 2007; Garden et al., 2002; Morris et al., 2008). Therefore, continued investigation in this area is warranted.

Interventions that could be used to train individuals to use an efficient semantic strategy during visuospatial working memory tasks could potentially boost performance and have real implications for quality of life and independent functioning. Importantly, semantic strategy training in other populations who typically exhibit lower working memory performance (e.g., mild cognitive impairment, dementia, depression), to our knowledge, remains to be investigated. However, strategy training is an important aspect of clinical neurorehabilitation following brain damage (see Wilson, 2023, for a review). A further important caveat, as mentioned previously, is that strategy use and instruction appear to depend on one’s ability to implement the intended strategy (Dunlosky & Kane, 2007; Nicholls & English, 2020; Nyberg et al. 2003, see also Pizzonia & Suhr, 2022, for a discussion on predictors of strategy use in older age). Thus, the wider implications and potential individual differences in the outcomes of strategy training require further scrutiny. Future research should consider incorporating more extensive task practice and/or using co-design methods for developing task instructions and interventions with the target population in order to ensure information is accessible and appropriate for the population (e.g., Slattery et al, 2020). Furthermore, an important future research aim should be to further clarify the strategies used across different age groups, and with other populations, ideally using more comprehensive approaches. This might include content and/or qualitative analyses of strategy reports (Ozimič et al., 2023), in addition to quantitative measurement throughout task performance.

### Is the use of semantics an automatic process or a strategic choice?

Empirically, semantics have been shown to support working memory task performance. This has been through both automatic processes, driven by the semantic properties of stimuli, and more active/strategic processing (Logie, 1995, 2003, 2011, 2023a). Indeed, there has been debate regarding the extent to which recruiting semantics to support working memory is an automatic process or a conscious, strategic choice (Gonthier, 2021). However, automatic and strategic processes may not be mutually exclusive. Semantics may be actively recruited to support encoding and maintenance of stimuli for working memory task performance, while the active use of semantics is under varying levels of conscious monitoring (Gonthier, 2021; Logie, 2011; Logie, Belletier et al., 2021). Indeed, in Baddeley and colleagues’ theoretical framework, the addition of the episodic buffer concept (Baddeley, 2000) addressed the need to incorporate the existence of a store that can draw information from both specialised working memory storage systems and long-term memory and bind these representations together in an integrated form. The episodic buffer can, therefore, create temporary bindings amongst abstract verbal and visuospatial stimuli and semantic representations in long-term memory. Originally, the episodic buffer was thought to be a principally passive system for maintaining integrated information. For example, the creation of bindings in the episodic buffer has been shown to function automatically, not requiring resources beyond those needed for storing single features (Allen et al., 2006). However, more recent work has suggested that ongoing executive control is important for prioritising items within the episodic buffer, where they are held in a privileged and consciously accessible state (Baddeley et al., 2021).

Importantly, more evidence is required to move towards making the distinction between automatic and active temporary bindings of working memory and long-term memory representations in more detail. Furthermore, a current question is whether temporary bindings of stimulus features require the concept of an episodic buffer as a single component rather than being a descriptive label for interactions between working memory and activated long-term memory (Logie, 2023a).

### Semantic contributions could hinder working memory performance

It is also important to note the bidirectional point of view in the literature, where semantic information can facilitate but also potentially impede memory performance. In some studies described previously, the use of semantic strategies did not significantly improve memory for items (e.g., Turley-Ames & Whitfield, 2003). Additionally, using semantic strategies can even hinder memory in some cases. A line of evidence demonstrating how concreteness of visual stimuli can impair recall concerns “verbal overshadowing.” In a classic study by Carmichael et al. (1932), providing verbal labels for abstract line drawings led to reproductions of the original stimuli that were less abstract and more closely represented the verbal labels. This demonstrates how concreteness of stimuli could be viewed as impairing recall of abstract stimuli (see also Hodel et al., 2022; Schooler & Engstler-Schooler, 1990). Furthermore, studies have found harmful influences of semantic long-term memory in the verbal domain using approaches including the two-list paradigm (e.g., Tehan & Humphreys, 1995). Following the encoding of a list of words, participants are asked to forget these words and encode a second list instead. Memory for the second list of words is tested using probed recall using a semantic category (e.g., animal) as the retrieval cue. When both lists contain words that match the retrieval cue (e.g., “dog” appeared in the first list and “cat” appeared in the second list), this can lead to the erroneous recall of words from the first list. In other words, the category cue can elicit the semantic representations of words from both lists, giving the opportunity for proactive interference to occur (Oberauer et al., 2017). Similarly, semantic distortions can occur in working memory tasks, creating false memories. For example, studies have shown that semantically related distractor stimuli can be falsely recognised during verbal working memory tasks (Rousselle et al., 2023; see also Roediger & McDermott, 1995; see Coane et al., 2021, for a review). These types of semantic errors can occur rapidly and confidently (Atkins & Reuter-Lorenz, 2008). However, Oberauer et al. found no evidence for proactive interference of long-term memory in visual working memory for objects and colours. In fact, previously learned object-colour associations induced knowledge that was only positive or neutral in terms of the impact on working memory task performance. Nevertheless, the possibility of reducing performance as a result of any memory task intervention should always be kept in mind, and such findings must always be reported and carefully considered.

## Conclusions

This review has explored theoretical links between working memory and long-term memory and the existing empirical evidence regarding the supportive use of semantics in working memory tasks. Working memory theories generally agree that activated long-term memory influences working memory performance. However, the precise way in which these memory processes or systems interact is less understood. A review of the existing empirical literature suggests that semantics may offer the opportunity to boost performance of various working memory tasks, across both the verbal and visuospatial domains. However, the precise ways in which semantics can be implemented to support working memory across different tasks requires further clarification (Gonthier, 2021). As an important caveat, a clearer, agreed-upon definition of semantics is required to correctly interpret semantic effects. However, it does appear that semantics may be recruited through automatic processes based on the semantic properties of stimuli, as well as more actively to support working memory via strategic approach (e.g., Brown & Wesley, 2013; Logie, 2011, 2023b). Given that semantics have been consistently associated with enhanced working memory performance and more efficient neural functioning, a critical avenue for future research is to investigate how semantic interventions could be developed and implemented to potentially maximise working memory performance, especially in the visuospatial domain in which research is extremely limited (Gonthier, 2021; Lemaire, 2016). This could potentially also extend to populations with lower working memory performance who may stand to benefit the most.
